# 

**Published:** 1899-10

**Authors:** 


### CONTENTS:

### SELECTIONS.

Article I.—A Mistake in Diagnosis. H. C. Gilchrist, D. D. S..... 241
" II.—Sterilization in Dental Practice. T. C. Van Kirk, D. D. S..... 243
" III.—A Dentifrice. Dr. W. J. Wallace..... 246
" IV.—Amalgam Pills. W. Andre Campbell, M. D. S..... 247
" V.—A Method of Pulp Canal Filling. G. W. Knight. D. D. S..... 249
" VI.—A New Method of Mummification of the Pulp. Professor Boennecken, M. D., D. D. S..... 252
" VII.—Reflex Dental Pain. S. F. Heverly, D. D. S..... 257
" VIII.—The Deciduous Teeth—Their Uses and Diseases. Dr. J. W. Juett, D. D S..... 259
" IX.—Local Anesthetics. J. S. Cassiday, D. D. S..... 262
" X.—Open Face Crown vs. Contour Filling or Richmond Crown for Incisors. H. T. King, D. D. S..... 265
" XI.—An Unusual Experience in Practice. I. B. Howell, D. D S..... 268
" XII—A Case of Tooth Impacted in the Left Bronchus: Gangrene of the Left Lung: Death..... 271
" XIII.—Rest and Sleep. J. Cam Anderson, M. D..... 273
" XIV.—The Marvels of Surgery..... 277

### MONTHLY SUMMARY.

A Mouthful of Teeth..... 281

Painless Extirpation of Live Pulps Without Cataphoresis..... 282

A Laboratory Hint..... 283

Teeth Made of Paper.—Latest Thing in Dentistry are Papier-Mache Molars..... 284

Refining Gold Scrap..... 285

Treatment of Porcelain Facings to Prevent Fracture..... 285

The Relation of Diseased Teeth to General Diseases..... 286

Causes of High Palate..... 287

Acidity of the Mouth During Sleep..... 288

Sodium Salicylate for Toothache..... 288

